# Prevalence and Characterization of Multi-Drug-Resistant Gram-Negative Bacilli Isolated From Lebanese Poultry: A Nationwide Study

**DOI:** 10.3389/fmicb.2018.00550

**Published:** 2018-03-23

**Authors:** Iman Dandachi, Elie S. Sokhn, Elias A. Dahdouh, Eid Azar, Bassel El-Bazzal, Jean-Marc Rolain, Ziad Daoud

**Affiliations:** ^1^Clinical Microbiology Laboratory, Faculty of Medicine and Medical Sciences, University of Balamand, Beirut, Lebanon; ^2^IRD, APHM, MEPHI, IHU-Méditerranée Infection, Aix-Marseille Université, Marseille, France; ^3^The Lebanese Ministry of Agriculture, Beirut, Lebanon

**Keywords:** ampC, ESBL, *E. coli*, poultry, carriage

## Abstract

Currently, antimicrobial resistance is one of the most prominent public health issues. In fact, there is increasing evidence that animals constitute a reservoir of antimicrobial resistance. In collaboration with the Lebanese Ministry of Agriculture, the aim of this study was to determine the prevalence of intestinal carriage of multi-drug-resistant Gram-negative Bacilli in poultry farms at the national level. Between August and December 2015, 981 fecal swabs were obtained from 49 poultry farms distributed across Lebanon. The swabs were subcultured on MacConkey agar supplemented with cefotaxime (2 μg/ml). Isolated strains were identified using MALDI-TOF mass spectrometry. Multilocus sequence typing analysis was performed for *Escherichia coli*. Phenotypic detection of extended spectrum β-lactamases (ESBL) and AmpC production was performed using double disk synergy and the ampC disk test, respectively. β-lactamase encoding genes bla_CTX-M_, bla_TEM_, bla_SHV_, bla_FOX_, bla_MOX_, bla_EBC_, bla_ACC_, bla_DHA_, and bla_CMY_ using PCR amplification. Out of 981 fecal swabs obtained, 203 (20.6%) showed bacterial growth on the selective medium. Of the 235 strains isolated, 217 were identified as *E. coli* (92%), eight as *Klebsiella pneumoniae* (3%), three as *Proteus mirabilis* (1%) and three as *Enterobacter cloacae* (1%). MLST analysis of *E. coli* isolates showed the presence of ST156, ST5470, ST354, ST155, and ST3224. The phenotypic tests revealed that 43.5, 28.5, and 20.5% of the strains were ampC, ESBL, and ampC/ESBL producers, respectively. The putative TEM gene was detected in 83% of the isolates, SHV in 20%, CTX-M in 53% and CMY ampC β-lactamase gene in 65%. Our study showed that chicken farms in Lebanon are reservoirs of ESBL and AmpC producing Gram-negative bacilli. The level of antibiotic consumption in the Lebanese veterinary medicine should be evaluated. Future studies should focus on the risk factors associated with the acquisition of multi-drug-resistant organisms in farm animals in Lebanon.

## Introduction

Antibiotic resistance is currently a major topic of interest for researchers and physicians. In particular, the rise of multi-drug resistance in Gram-negative bacteria is now a serious challenge encountered by healthcare professionals (Exner et al., 2017). Resistance in Gram-negative bacteria is mainly mediated via the production of extended spectrum β-lactamases (ESBL), ampC β-lactamases and carbapenemases (Schill et al., 2017). Genes encoding these enzymes are often located on plasmids carrying resistance genes to other commonly used antibiotics in clinical settings (Seiffert et al., 2013). Infections with these multi-drug-resistant organisms (MDROs) will thus pose therapeutic challenges; the antibiotic pipeline is drying up, and no new antimicrobial agents are anticipated in the near future to treat infections caused by MDROs (Bettiol and Harbarth, 2015).

In fact, it has been generally accepted that the main driver for the rapid evolution of bacterial resistance is the uncontrolled usage of antibiotics in human medicine. It is suggested that this theory is also applicable to the veterinary sector (Kempf et al., 2015). The European Centre for Disease Prevention and Control/European Food Safety Authority/European Medicines Agency (ECDC/EFSA/EMA) joint report stated that in 2014, the average antibiotic consumption in animals (152 mg/kg) was higher than in humans (124 mg/kg). Univariate analysis showed a signification correlation between fluoroquinolone consumption and resistance in *Escherichia coli* in the human and animal sectors, between polymyxins and tetracyclines and *E. coli* in animals, and for 3rd/4th generation cephalosporins and *E. coli* in humans (ECDC/EFSA/EMA, 2017). Antibiotics are heavily administered for therapeutic and prophylaxis purposes in veterinary medicine. As growth promoters, this practice is no longer adapted in the European Union, whereas it persists in North America and other countries (Economou and Gousia, 2015). In their study, Chantziaras et al. (2014) found a significant correlation between the use of antibiotics in livestock and the corresponding level of resistance toward these antimicrobials in *E. coli* strains isolated from pigs, poultry and cattle. During the last years, the prevalence of ESBLs, ampC, and carbapenemase producing Gram-negative bacteria has become extensively reported in food producing animals (Ghodousi et al., 2015; Gonzalez-Torralba et al., 2016; Haenni et al., 2016). In their review paper, Schwarz et al. (2016) showed that studies describing the epidemiology of resistant organisms in livestock targeted mainly swine, cattle and poultry. The prevalence of resistance varied from one country to another (Alonso et al., 2017). Although the extent to which food of animal origin contributes to the zoonotic transmission of multi-drug-resistant organisms, i.e., ESBL and carbapenemase producers, has not yet been well established (Madec et al., 2017), it suggests that sharing the same ESBL genes, plasmids and strains constitutes possible evidence of zoonotic transmission of MDROs from animals to humans (Leverstein-van Hall et al., 2011; Dahms et al., 2014). Furthermore, the increased risk of ESBL fecal carriage in individuals with a high degree of contact with broiler chickens is an indicator of transmission (Huijbers et al., 2014). Enteric-resistant strains in livestock can be easily transferred to humans through direct contact or through the handling/consumption of undercooked/uncooked animal products (Dahms et al., 2014).

In Lebanon, several studies addressing MDROs in hospital settings have been conducted. One study done at the American University of Beirut Medical Center between 2008 and 2011 reported that 1.07 and 2.45% of *E. coli* and *Klebsiella pneumoniae* clinical isolates, respectively, were ESBL producers and ertapenem-resistant (Baroud et al., 2013). Another study conducted in the north reported that over the period of 2009–2012, 9% and 28% of the bacteraemia episodes in febrile neutropenic patients were caused by carbapenem and third-generation cephalosporin-resistant Gram-negative bacilli, respectively (Moghnieh et al., 2015). However, very few studies have addressed this issue in the environment. One study showed that *Acinetobacter baumannii* was detected in 6.9% of water samples, 2.7% of milk samples, 8.0% of meat samples, 14.3% of cheese samples and 7.7% of animal samples (Rafei et al., 2015). Another study in which 115 stool samples were collected from livestock animals from different farms in north Lebanon reported the detection of four VIM-2 producing *Pseudomonas aeruginosa*, four OXA-23 producing *A. baumannii* and one OXA-23/OXA-58 co-producing *A. baumannii* (Al Bayssari et al., 2015a). Furthermore, Al Bayssari et al. (2015b) reported the isolation of an OXA-48 harboring *E. coli* isolate from fowl in Lebanon. More recently, Diab et al. (2016) detected a relatively high prevalence of CTX-M-15 producing *E. coli* in Lebanese cattle. In the above-mentioned studies in Lebanese livestock, MLST analysis revealed the presence of sequence types common to both humans and animals (Al Bayssari et al., 2015a; Rafei et al., 2015; Diab et al., 2016), which suggests that Lebanese farms are potent reservoirs of multi-drug-resistant organisms that could be transmitted to humans. In the present study and in collaboration with the Lebanese Ministry of Agriculture, our aim was to determine the national epidemiology of multi-drug-resistant Gram-negative bacilli in Lebanese chicken farms in terms of intestinal carriage.

## Materials and Methods

### Ethics Statement

The Ministry of Agriculture in Lebanon granted approval to collect chicken samples from representative farms in the country as per the national norms for animal sampling and manipulation. This sampling was in conformity with the international regulations for animal safety. All of the involved farms officially received authorization from the Ministry of Agriculture, and this was considered, after undergoing an acceptance process, an official and legal document. Therefore, an Institutional Review Board (IRB) approval was obtained for the present study.

### Samples Collection

Between August and December 2015, 981 rectal swabs were collected from 49 poultry farms distributed over the seven districts of Lebanon. Six to seven farms were visited in each district. The average number of samples taken from each farm was 20 fecal swabs (**Table 1**). The 20 samples collected were randomly taken from each farm. Technical assistance, i.e., fecal swabs, gloves, costumes, and a portable refrigerator, were provided by the Ministry of Agriculture team. The collected swabs were directly placed in a portable refrigerator, and when they arrived at the University Laboratory, they were stored at -80°C until use. The farms visited were selected by considering their geographical location, presence or absence of a nearby community and the size of the farms (at least 3,000 chickens per breeding site). Eighty percent of the samples were gathered from broiler chickens, while 20% were taken from layers. The mean average age of the broilers and layers was 31 days and 14 months, respectively.

**Table 1:** Distribution of MDROs per farm and district.

		Collection date	Farm size	Age	Type	# of collected samples	# of positive samples	# of isolated strains
	Fl		18000	35 d	B	27	11	11
	F2		11300	35 d	B	27	5	6
	F3		20000	45 d	B	27	2	2
North Leb	F4	27-Aug	23000	4 m	L	20	9	18
	F5		4000	35 d	B	20	14	23
	F6		20000	25 d	B	20	13	14
	F7		15000	35 d	B	20	8	9
	F8		5000	25 d	B	20	5	5
Akkar	F9	31-Aug	4000	25 d	B	20	5	5
	F10		6000	25 d	B	20	9	11
	F11		4600	4 m	L	20	11	14
	F12		15000	40 d	B	20	11	14
	F13		6000	45 d	B	20	1	1
	F14		10700	36 d	B	20	4	4
Bekaa	F15	15-Sep	5000	45 d	B	20	6	7
	F16		3000	18 m	L	20	3	3
	F17		6000	36 d	B	20	1	1
	F18		6000	43 d	B	20	6	7
	F19		6000	43 d	B	20	3	3
Baalbek	F20	21-Sep	5000	14 m	L	20	3	3
	F21		6500	27 d	B	20	3	3
	F22		6700	12 m	L	21	1	1
	F23		11800	26 d	B	20	4	4
Nabatieh	F24	21-Oct	10000	27 d	B	20	2	2
	F25		10000	25 d	B	20	1	1
	F26		5000	25 d	B	20	1	1
	F27		10000	27 d	B	20	8	8
	F28		5000	28 d	B	20	4	4
Jabal Leb	F29	9-Nov	5000	25 d	B	20	7	6
	F30		10000	27 d	B	20	2	2
	F31		10000	28 d	B	20	4	5
	F32		18000	25 d	B	20	5	5
	F33		6000	25 d	B	20	3	3
	F34		6000	25 d	B	20	6	6
Saida	F35	7-Dec	3300	32 d	B	20	10	10
	F36		10000	25 d	B	20	5	6
	F37		10000	30 d	B	20	1	1
	F38		10000	28 d	B	20	6	6

*F, farm; Aug, August; Sept, September; Oct, October; Nov, November; Dec, December; d, days; m, month; B, broiler; L, layer.*

### MALDI-TOF MS Identification

Rectal swabs were sub-cultured on a MacConkey agar supplemented with 2 μg/ml of cefotaxime for the preliminary screening of antibiotic-resistant Gram-negative bacilli. After overnight incubation at 37°C, colonies showing different morphologies were picked up from each selective plate and tested separately with MALDI-TOF MS for identification using the Microflex LT spectrometer (Bruker Daltonics, Bremen, Germany) (Seng et al., 2010; Singhal et al., 2015). The spectra obtained for each strain were stored and downloaded into a MALDI Biotyper 3.0 system to create a single main spectrum for each bacterial isolate. Thereafter, a dendrogram was constructed using MALDI Biotyper 3.0 software.

### Antibiotic Susceptibility Testing

Using the Kirby–Bauer disk diffusion method, antibiotic susceptibility testing was performed. The results were interpreted according to EUCAST guidelines 2017 (European Committee on Antimicrobial Susceptibility Testing, 2017). Sixteen antimicrobial agents were used including ampicillin, aztreonam, cefotaxime, ceftazidime, cefoxitin, cefepime, amoxicillin-clavulanic acid, piperacillin-tazobactam, meropenem, imipenem, ertapenem, colistin, tigecycline, ciprofloxacin, gentamicin and trimethoprim-sulfamethoxazole (Bio-Rad, Marnes-la-Coquette, France). Phenotypic detection of ESBL was performed using the double-disk synergy test by placing an amoxicillin–clavulanic acid disk in the center between aztreonam, cefepime and ceftazidime. The observation of a “key hole effect” was considered a positive test. On the other hand, ampC β-lactamase detection was performed using the ampC disk test (Black et al., 2005). In brief, a lawn of cefoxitin-susceptible *E. coli* ATCC 25922 was inoculated on the surface of a Mueller Hinton agar plate. A 30-μg cefoxitin disk was placed on the inoculated surface. A sterile filter paper disk was moistened by adding 20 μl of a 1:1 mixture of saline and 100 × Tris-EDTA (catalog code T-9285; Sigma-Aldrich Corporation, St. Louis, MO, United States). Several colonies of the test isolate were then applied to the disk. The disk was then positioned with its inoculated face in contact with the agar surface. After overnight incubation, a flattening or indentation of the zone of inhibition around the cefoxitin disk was considered a positive result, while an absence of distortion was considered a negative one. Furthermore, for the presumptive detection of carbapenemases, the carba NP test was performed as previously described (Bakour et al., 2015). A bacterium was characterized as being multi-drug-resistant when resistance to at least three classes of antibiotics was observed (Magiorakos et al., 2012).

### Molecular Characterization of β-Lactamase Encoding Genes

All of the isolates that showed a key hole effect or had cefoxitin resistance with non-susceptibility to cefepime were subjected to real-time PCR analysis for the detection of SHV, TEM and CTX-M encoding genes (Roschanski et al., 2014). Simplex PCRs for the genes encoding AmpC β-lactamases FOX, MOX, ACC, EBC, DHA, and CMY were conducted for all strains showing non-susceptibility to cefoxitin (Dallenne et al., 2010). Simplex PCR was also used to test the ADC ampC β-lactamase gene in *A. ba*umannii (Liu and Liu, 2015). DNA extraction was performed according to the manufacturer’s instructions using EZ1 DNA extraction kits (Qiagen, Courtaboeuf, France) with the EZ1 Advanced XL biorobot.

### Multilocus Sequence Typing

One *E. coli* strain from each cluster shown in the MSP dendrogram was chosen, and MLST typing was performed based on allelic profiles to determine their evolutionary relationship (Peng and Zong, 2011). Seven housekeeping genes were used*: adk, fumC, gyrB, icd, mdh, purA*, and *recA*. Analysis of the genes’ allelic profiles was performed on the MLST^1^ to determine the sequence type (ST) to which each isolate belongs.

### Statistical Analysis

The prevalence, identification, and resistance profiles of isolated strains are all presented as the number (percentage).

## Results

### Bacterial Identification

**FIGURE 1:**
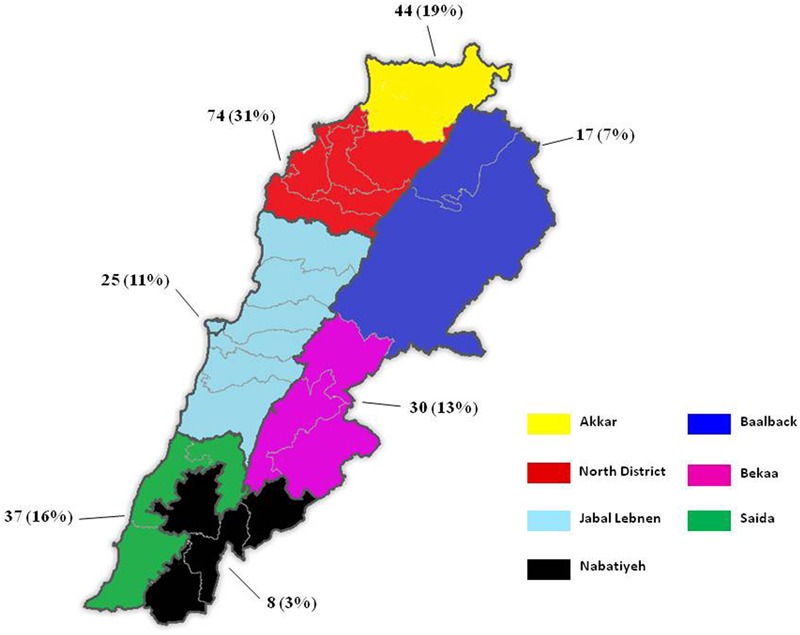
MSP dendrogram of *Escherichia coli* isolates.

Out of 982 collected fecal swabs, 203 (20.6%) showed growth on selective medium. In total, 235 strains were isolated. All 235 isolated Gram-negative bacilli were identified by MALDI TOF mass spectrometry with a score value ≥1.9. The distribution at the species level was as follows: 217 were identified as *E. coli* (92%), eight as *K. pneumoniae* (3%), three as *Proteus mirabilis* (1%), three as *Enterobacter cloacae* (1%), two as *E. albertii*, one as *E. fergusonii* and one as *A. baumannii*. The MSP dendrogram of the 217 *E. coli* isolates revealed five clusters at a distance level of 500 (arbitrarily selected) (**Figure 1**). Cluster 1 was mainly formed by isolates from the Akkar District. Cluster 2 contained two isolates: one from Saida and the other from Baalbek. Cluster 3 was composed of three strains isolated from Jabal Lebnen District. Cluster 4 was mainly composed of isolates from the North Lebanon district, and Cluster 5 contained only one strain from Saida.

### Phenotypic Profiles of Resistance

The disk diffusion susceptibility testing results are summarized in **Table 2**. All of the isolates were susceptible to tigecycline, colistin and carbapenems. Phenotypic identification using the double disk synergy test, ampC disk test and carba NP test revealed that 102 (43.5%) of the isolated strains were ampC β-lactamase producers, 67 (28.5%) were ESBL producers, and 48 (20.5%) were co-producers of ESBL and ampC β-lactamases. Both ESBL and ESBL/ampC production were detected in *E. coli, K. pneumoniae, E. fergusonii*, and *E. cloacae* (**Table 2**), whereas only AmpC production was detected in *E. coli, K. pneumoniae, P. mirabilis, E. albertii*, and *A. baumannii*. In addition, 18 *E. coli* strains (7.5%) did not show a key hole effect and were resistant to cefoxitin but tested negative with the ampC disk test. Moreover, 32% of the isolated strains were co-resistant to gentamicin, ciprofloxacin, and trimethoprim-sulfamethoxazole, whereas 40% were resistant to at least two non-β-lactam antibiotics, 19.5% were resistant to only one non-β-lactam, and 8% were susceptible to all of the non-β-lactam antibiotics tested.

**Table 2:** Resistance profiles and phenotypes of multi-drug-resistant organisms isolated in this study.

Species	AMP	AZT	CTX	CAZ	FOX	FEP	AMC	TZP	SXT	CIP	GENT	% of ESBL producers	% of AmpC producers	% of ESBL/AmpC co-producers
*Escherichia coli* (*n* = 217)	217 (100)	49 (23)	195 (90)	120 (55)	104 (48)	31 (14)	77 (35)	28 (13)	150 (59)	134 (62)	152 (70)	27	44	21
*Klebsiella pneumonia* (*n* = 8)	8 (100)	2 (25)	8 (100)	3 (38)	2 (25)	2 (25)	2 (25)	2 (25)	6 (75)	7 (88)	7 (88)	50	37.5	12.5
*Proteus mirabilis* (*n* = 3)	3 (100)	0 (0)	2 (67)	0 (0)	3 (100)	0 (0)	3 (100)	0 (0)	3 (100)	3 (100)	1 (33)		100	
*Enterobacter cloacae* (*n* = 3)	3 (100)	1 (33)	3 (100)	2 (67)	0 (0)	0 (0)	0 (0)	0 (0)	1 (33)	1 (33)	3 (100)	100		
*Escherichia albertii* (*n* = 2)	2 (100)	0 (0)	1 (50)	1 (50)	2 (100)	0 (0)	0 (0)	0 (0)	0 (0)	0 (0)	0 (0)		100	
*Escherichia fergusonii* (*n* = l)	1 (100)	0 (0)	1 (100)	0 (0)	0 (0)	0 (0)	0 (0)	0 (0)	0 (0)	1 (100)	0 (0)	100		
*Acinetobacter baumannii* (*n* = l)	1 (100)	0 (0)	1 (100)	1 (100)	1 (100)	0 (0)	0 (0)	0 (0)	0 (0)	0 (0)	0 (0)		100	

*Resistance profiles are presented as a number (percentage). N, number; %, percentage; AMP, ampicillin; AZT, aztreonam; CTX, cefotaxime; CAZ, ceftazidime; FOX, cefoxitin; FEP, cefepime; AMC, amoxicillin-clavulanic acid; TZP, piperacillin-tazobactam; SXT, trimethoprim-sulfamethoxazole; CIP, ciprofloxacin; GENT, gentamicin.*

### Prevalence of MDR-GNB

**FIGURE 2:**
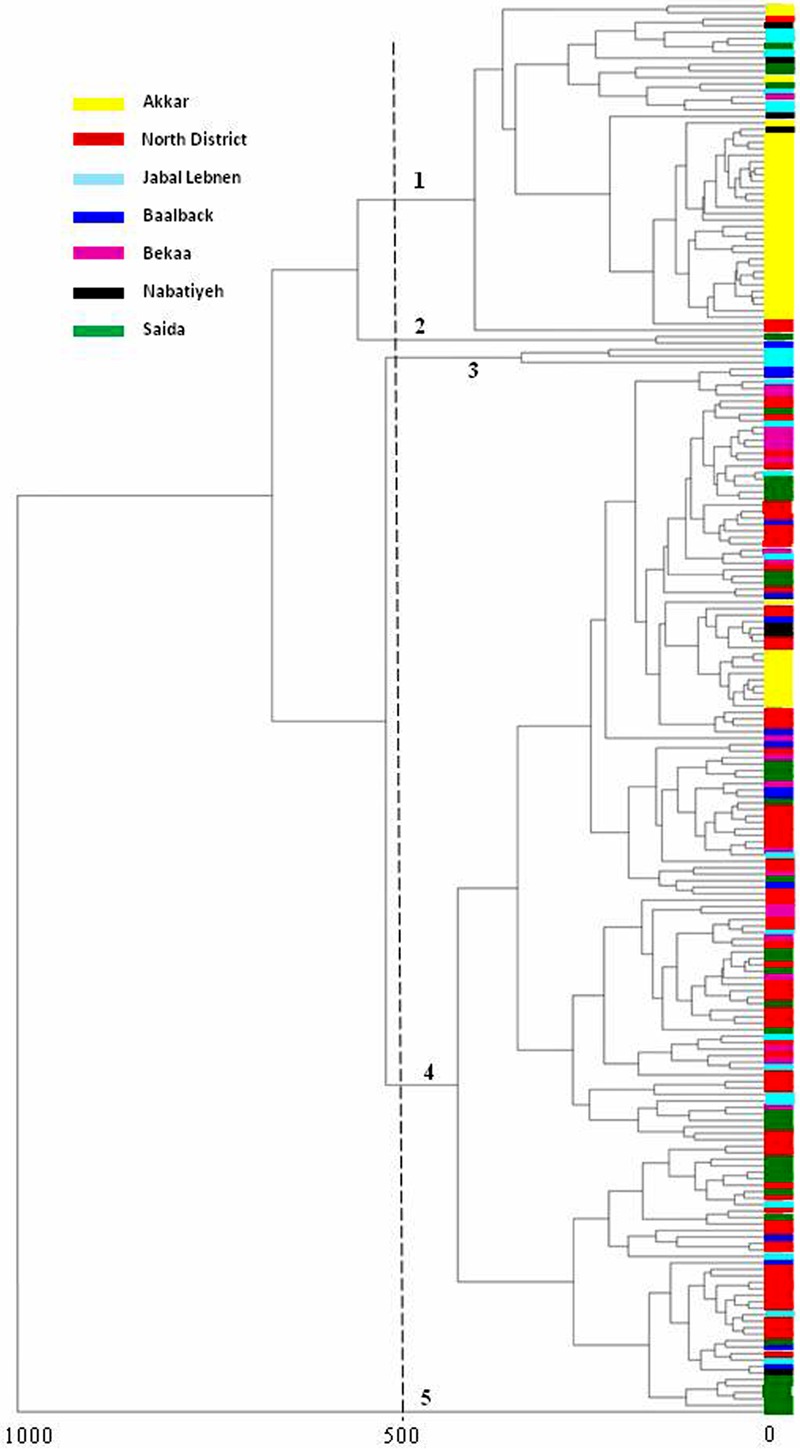
Prevalence of MDROs in Lebanese poultry farms. Prevalence is expressed as the “number of isolates (percentage).”

The distribution of samples showing positive growth on the selective medium was as follows: 54 samples in the North District, 38 in the Akkar District, 37 in Saida, 26 in Bekaa, 24 in Jabal Lebnen, 16 in Baalbek and eight in Nabatieh. The number of positive samples from broilers exceeded the one obtained from Layers (176 vs. 27, respectively). Isolated strains (235) originated from 38 out of the 49 visited farms, i.e., 77.5% of the farms were positive for at least one multi-drug-resistant Gram-negative bacilli. As shown in **Figure 2**, the highest prevalence was detected in the north-west of the country, with 74 and 44 isolated strains for the North and Akkar Districts, respectively, whereas the lowest prevalence was detected in the north–east and south–east of Lebanon.

### PCR Screening of CTX-M, SHV, TEM, and AmpC β-Lactamase Genes

One hundred and twelve isolates suspected to be ESBL producers were subjected to a real-time PCR assay for the detection of SHV, TEM, and CTX-M encoding genes. Of the 112 strains selected, 93 (83%) harbored the TEM gene, 59 (53%) the CTX-M gene and 22 (20%) the SHV gene. Overall, 49% (55) of the ESBL suspected isolates harbored only one gene, 46% (52) harbored at least two genes with the highest concordance being between the TEM and CTX-M genes, and 4% (five) showed the co-existence of all three genes together (**Table 3**). In parallel, 152 strains including 4 *K. pneumoniae*, 3 *P. mirabilis*, 2 *E. albertii*, and 143 *E. coli* were positive for *bla*_CMY_; whereas fifteen *E. coli* strains were negative fall ampC β-lactamase genes tested. Furthermore, in *A. baumannii* the ADC gene was detected.

**Table 3:** Characteristics of the different phenotypes/genotypes of ESBL and ESBL/AmpC producers found in this study.

Species	Phenotype	β -lactamase genes			Co-resistance to non β -lactams
*Escherichia coli*	ESBL		bla TEM	bla CTX-M	SXT-CIP-GNT
			bla TEM	bla CTX-M	SXT-CIP
			bla TEM	bla CTX-M	CIP-GNT
			bla TEM	bla CTX-M	SXT-GNT
		bla SHV	bla TEM		SXT-CIP-GNT
		bla SHV	bla TEM		CIP
		bla SHV	bla TEM		SXT-GNT
		bla SHV	bla TEM		SXT-CIP
		bla SHV	bla TEM		SXT
				bla CTX-M	SXT-CIP-GNT
				bla CTX-M	SXT-CIP
				bla CTX-M	N.R
			bla TEM		SXT-CIP-GNT
			bla TEM		SXT-GNT
			bla TEM		SXT-CIP
			bla TEM		CIP-GNT
			bla TEM		GNT
			bla TEM		N.R
		bla SHV	bla TEM	bla CTX-M	SXT-CIP-GNT
		bla SHV			GNT
	AmpC/ESBL		bla TEM		SXT-CIP-GNT
			bla TEM		SXT-GNT
			bla TEM		CIP-GNT
			bla TEM		SXT
			bla TEM		N.R
			bla TEM	bla CTX-M	SXT-CIP-GNT
			bla TEM	bla CTX-M	SXT
			bla TEM	bla CTX-M	CIP-GNT
			bla TEM	bla CTX-M	SXT-CIP
			bla TEM	bla CTX-M	SXT-GNT
			bla TEM	bla CTX-M	N.R
		bla SHV	bla TEM		GNT
		bla SHV	bla TEM		CIP-GNT
				bla CTX-M	SXT-CIP-GNT
				bla CTX-M	N.R
		bla SHV		bla CTX-M	CIP-GNT
		bla SHV	bla TEM	bla CTX-M	SXT-CIP-GNT
*Klebsiella pneumoniae*	ESBL	bla SHV	bla TEM		SXT-CIP-GNT
		bla SHV	bla TEM	bla CTX-M	SXT-CIP-GNT
			bla TEM	bla CTX-M	CIP-GNT
	AmpC/ESBL	bla SHV	bla TEM	bla CTX-M	SXT-CIP-GNT
			bla TEM	bla CTX-M	SXT-CIP-GNT
		bla SHV	bla TEM		SXT-GNT
*Escherichia fergusonii*	ESBL		bla TEM	bla CTX-M	CIP
*Enterobacter cloacae*	ESBL			bla CTX-M	GNT

*SXT, trimethoprim-sulfamethoxazole; GNT, gentamicin; CIP, ciprofloxacin; N.R, no resistance.*

### MLST Typing

The MLST typing of the strains, each chosen from the major district-related isolates grouped in each cluster, revealed that they belong to five different STs: ST156 for Cluster 1, ST5470 for Cluster 2, ST354 for Cluster 3, ST155 for Cluster 4 and ST3224 for Cluster 5.

## Discussion

Many years ago, hospitals and health care settings were regarded as the sole source of antimicrobial resistance. However, recent evidence has shown that food producing animals constitute a potent reservoir of multi-drug-resistant organisms (Belmahdi et al., 2016; Bachiri et al., 2017). This was mainly linked to the over-use of antimicrobial agents in veterinary medicine for treatment, growth promotion and prophylaxis (Economou and Gousia, 2015). Although the zoonotic transmission of multi-drug-resistant organisms from animals to humans remains controversial (Olsen et al., 2014), several studies have shown a direct link between direct contact with farm animals and the acquisition of bacterial resistance (Huijbers et al., 2014). One study conducted by Olaitan et al. (2015) demonstrated the zoonotic transmission of a colistin-resistant *E. coli* strain from a pig to its owner. This owner usually fed his pig without wearing any protective equipment. The two colistin-resistant isolates (in the pig and its owner) belonged to the same sequence type and presented with the same virulence and PFGE pattern (Olaitan et al., 2015).

In Lebanon, very few studies have looked at the prevalence of MDROs in farm animals (Al Bayssari et al., 2015a). Our study is the first epidemiological study in Lebanon quantifying the prevalence of multi-drug-resistant Gram-negative bacilli in chicken farms in terms of intestinal carriage at the national level. The prevalence is similar to the one previously reported from cattle (84%) in Lebanon (Diab et al., 2016). The flock’s size did not influence the prevalence of resistance in each farm (**Table 1**). On a global level, the prevalence found in our study is approximate to the one reported in Romania (69%) (Maciuca et al., 2015) and Ecuador (60%) (Ortega-Paredes et al., 2016) but is higher than the ones described in Germany (44%) (Kola et al., 2012), Japan (23%) (Kawamura et al., 2014), and Vietnam (3.2%) (Nguyen et al., 2015). Differences in the screening methodologies, sample size used and the level of antibiotic consumption in each country could explain these variations (Rhouma et al., 2016).

*Escherichia coli* was the most common multi-drug-resistant organism isolated; MALDI-TOF MSP dendrogram and MLST analysis revealed the presence of five clusters from which the representative strains belonged to different STs. Within each cluster, strains isolated from farms of the same district were grouped together; this is especially true for the Akkar and North Lebanon strains. This observation reveals that strains of the same region are closely related. Although PFGE is the standard method for the detection of clones, due to the large number of strains isolated in this study, PFGE typing was not performed; rather, we referred to the MSP dendrogram as a possible rapid tool for strain differentiation according to their geographical and/or phenotypic distribution in epidemiological studies as certain previous studies have suggested (Berrazeg et al., 2013; Khennouchi et al., 2015). With the exception of ST155, none of the sequence types identified in this study were among those frequently reported in chicken such as ST10, ST23, ST48, ST58, ST115, ST117, ST350, and ST648 (Olsen et al., 2014). However, looking at the Warwick *E. coli* MLST database, we found that the STs detected in our study were previously reported from livestock, cats and dogs, and humans. ST155 has been commonly reported in poultry (Pires-dos-Santos et al., 2013), and it appears to be associated with a zoonotic risk, which has been suggested by some studies (Lazarus et al., 2015). This emphasizes the hypothesis that MDROs in food-producing animals can be transmitted to humans and may be causative agents of infections with therapeutic challenges when high resistance is encountered. It should also be mentioned that clones in animals and humans are not always shared; some studies have shown that *E. coli* strains in food-producing animals differ from those reported in humans (Randall et al., 2012; Wu et al., 2013). This suggests that only some bacterial clones might be transmitted to the human population.

As our study showed, ESBL producers dominate the Lebanese poultry sector. The prevalence of ampC producers is also elevated (43.5%). ESBL and ampC-producing Gram-negative bacilli were previously reported in clinical and community settings in Lebanon (Dandachi et al., 2016). Molecular characterization revealed that 50% of isolated strains co-harbored at least two β-lactamase genes with the most common being CTX-M and TEM. Moreover, the only AmpC β-lactamase encoding gene was the CMY ampC β-lactamase. This gene was previously reported in poultry (Dierikx et al., 2013; El-Shazly et al., 2017) as well as in food producing animals (Sato et al., 2014; Aguilar-Montes de Oca et al., 2015) and healthy pets (Donati et al., 2014; Liu et al., 2016). As per the phenotypic and genotypic detection of AmpC production, these showed that there are some strains that were negative with the ampC disk test but positive for an ampC β-lactamase gene and vice-versa. Phenotypically false negatives shows the importance of the molecular testing in the detection of AmpC production. On the other hand, in the 15 *E. coli* strains that were negative for plasmidic ampC β-lactamase genes; one explanation for this might be due to an overexpression of the chromosomal ampC gene mediated by a mutation in the promoter/attenuator region as described in previous studies (Escudero et al., 2010; Haenni et al., 2014). Regarding non-β-lactam co-resistance in ESBL and/or ampC producers, antimicrobial resistance toward gentamicin was relatively high in this study. In fact, 66% of ESBL and/or ampC producing Gram-negative bacilli were gentamicin resistant. This could possibly be linked to the frequent use of this antibiotic in Lebanese farms as several studies have reported (El-Rami et al., 2012; Diab et al., 2016). One study conducted by Abdelnoor et al. (2013) found a significant association between gentamicin resistance in *E. coli* isolates and the use of this antimicrobial agent as a food additive in poultry in Lebanon. Another study launched a questionnaire-based survey on the most common antibiotics used in Lebanese livestock and found that gentamicin and streptomycin are the most common and heavily used antimicrobial agents (Kassaify et al., 2013). Another thing to mention is that in this study, no carbapenemase producers were detected. There might be two possible explanations for this: the first one is that carbapenemase producers are really scarce in Lebanese chicken farms; the second one is that these isolates were missed due to the medium used for the screening of multi-drug-resistant organisms. As has been reported, OXA-48 carbapenemase producers are frequently found in hospitals and nursing homes and in fowls in Lebanon (Al Bayssari et al., 2015b). OXA-48 carbapenemases do not always confer resistance to third-generation cephalosporins unless there is another mechanism of resistance that co-exists in the same bacterial cell (Poirel et al., 2012). Therefore, Oxacillinase producers could have been missed or under-estimated in our study.

Our study has two main limitations. The first one is that the primers used for blaTEM and blaSHV screening were universal, and thus, the possibility of having non-ESBL variants cannot be ruled out. However, as the strains presented with a typical ESBL phenotype, i.e., the key hole effect and resistance to penicillin, monobactams and third-generation cephalosporins with susceptibility to carbapenems, the TEM-positive strains were considered as ESBL producers and were included in the description of the MDR-GNB prevalence in this study. The second limitation is the low number of isolates subjected to MLST typing. MLST and PFGE analysis remain the gold standard for clone/cluster detection in epidemiological studies regardless of the number of strains (McGregor and Spratt, 2005; Zou et al., 2010).

## Conclusion

Our study illustrates the current epidemiology of multi-drug-resistant Gram-negative bacilli in Lebanese chicken farms. ESBL and ampC producers cross-resistant to antibiotics used in human medicine are highly prevalent across the territory. Our study suggests that poultry farms are potent reservoirs of antimicrobial resistance in Lebanon. Although very few studies have reported the detection of carbapenemase producers in Lebanese Livestock (Al Bayssari et al., 2015a,b), it will likely only be a matter of time before these organisms become prevalent in Lebanese animal farms. This is especially true if no strict rules are implemented to control the overuse and misuse of antibiotics for treatment, growth promotion and prophylaxis in Lebanese agriculture. We believe that the prescription of antibiotics often used in human medicine should be reduced or even banned in the veterinary sector.

## Author Contributions

ID, ES, and ED conducted the phenotypic and molecular work. BE-B was responsible for the collection of the samples. EA, J-MR, and ZD reviewed and edited the manuscript.

## Conflict of Interest Statement

The authors declare that the research was conducted in the absence of any commercial or financial relationships that could be construed as a potential conflict of interest.

## Footnotes

1 http://mlst.warwick.ac.uk/mlst/dbs/Ecoli
